# Disc degeneration on MRI is more prevalent in young elite skiers compared to controls

**DOI:** 10.1007/s00167-017-4545-3

**Published:** 2017-04-13

**Authors:** Wisam A. Witwit, Peter Kovac, Anna Sward, Cecilia Agnvall, Carl Todd, Olof Thoreson, Hanna Hebelka, Adad Baranto

**Affiliations:** 1 Department of Radiology, Institute of Clinical Sciences at Sahlgrenska Academy, University of Gothenburg and Sahlgrenska University Hospital, 41345 Gothenburg, Sweden; 2 Department of Orthopaedics, Institute of Clinical Sciences at Sahlgrenska Academy, University of Gothenburg and Sahlgrenska University Hospital, Gothenburg, Sweden; 3 Sportsmedicine Åre and Åre Ski High School, Ostersund, Sweden

**Keywords:** Disc degeneration, Schmorl’s nodes, Athletes, Skiers, MRI

## Abstract

**Purpose:**

Evidence-based facts regarding spine abnormalities and back pain are needed in order to develop rehabilitation programs and prevent spine injuries in young skiers. The aim therefore is to identify MRI changes in the thoraco-lumbar spine and the lifetime prevalence of back pain, as well as the association between them, in young skiers compared to non-athletes.

**Methods:**

Seventy-five young elite alpine and mogul skiers, age range 16–20 years, were compared with 27 non-athletic controls. All subjects underwent spinal MRI and answered back pain questionnaires.

**Results:**

Fifty-six percent of skiers had at least one disc Pfirrmann grade ≥3 compared to 30% of controls (*p* = 0.027). Schmorl’s nodes (46%) and disc height reduction (37%) were significantly more prevalent in skiers compared to controls (0%) (*p* < 0.001). When all parameters were combined together, skiers had significantly higher rate of radiological changes than controls, 82% compared to 54% (*p* = 0.007). The mean number of discs with Pfirrmann grade ≥3 was 1.1 per individual in skiers (median 1, range 0–6) versus 0.6 in controls (median 0, range 0–3). There was no significant difference in lifetime prevalence of back pain between skiers (50%) and controls (44%) (n.s.). MRI abnormalities in skiers did not correlate with lifetime prevalence of back pain. Skiers had a better health perception than controls (*p* = 0.026).

**Conclusion:**

Alpine skiers have more degenerative disc changes compared to non-athletes, but these changes do not correlate with the lifetime prevalence of back pain. Lifetime prevalence of back pain is not significantly different between the groups; however, skiers report more severe pain on VAS score.

**Level of evidence:**

II.

## Introduction

The increased interest in physical exercise and participation in sports among adolescents has resulted in a trend to begin training, competing, and specializing in one sport at a very young age. Nowadays, it is a common opinion that high doses of training, for long periods of time, are required at already a young age in order to become an elite athlete [2, 17].

In athletes with great demands on the spine, such as wrestlers, gymnasts, weightlifters, divers, ice-hockey, soccer- and tennis players, a high rate of radiological changes has been reported in the thoraco-lumbar spine [4, 5, 12, 13, 16, 18, 39, 40]. Examples of such changes are disc degeneration, disc herniation, apophyseal ring injury and pars interarticularis fractures. The incidence of these findings has been reported to be higher during growth spurt [4, 5, 12, 14, 20, 34, 37, 41, 42].

Alpine skiing is a high-risk sport regarding trauma and overloading injuries with the immature spine being even more vulnerable [1, 9, 10, 26]. Overuse injuries are mostly common in the back of alpine skiers, and this is believed to be correlated with the repetitive loading the skiers are subjected to [38]. A significantly high rate of spinal abnormalities has been identified on plain radiographs of young skiers [29, 33]. A high prevalence of back pain has also been reported in high school alpine skiers, especially after training progression [6]. On the contrary, lifetime prevalence of back pain in ski instructors has been reported to be similar to the general population [31].

In epidemiological studies and systematic reviews, variable results have been published regarding back pain prevalence and its association with excessive physical exercise in young athletes [23, 29, 33]. In a review for the years 1951 until 2013, back pain was found to be a common complaint in athletes with both spinal and extra-spinal causal factors identified such as disc degeneration, spondylolisthesis, disc herniation, apophyseal ring injury, sacral stress fracture, muscle injuries and/or ligament tears [6, 7, 28, 31]. The association of spinal abnormalities, if any, with back pain is still not fully elucidated.

Therefore, more studies are needed to establish evidence-based facts concerning spine abnormalities and back pain in order to develop rehabilitation programs and prevent spine injuries in young skiers. Previous studies are based mainly on the analysis of plain radiographs [29, 33] while MRI has a higher sensitivity and specificity regarding spinal abnormalities and it is assumed to detect spinal changes earlier than plain radiographs [35]. Also, there are no prior studies regarding back pain and MRI changes in mogul skiers.

**Fig. 1:**
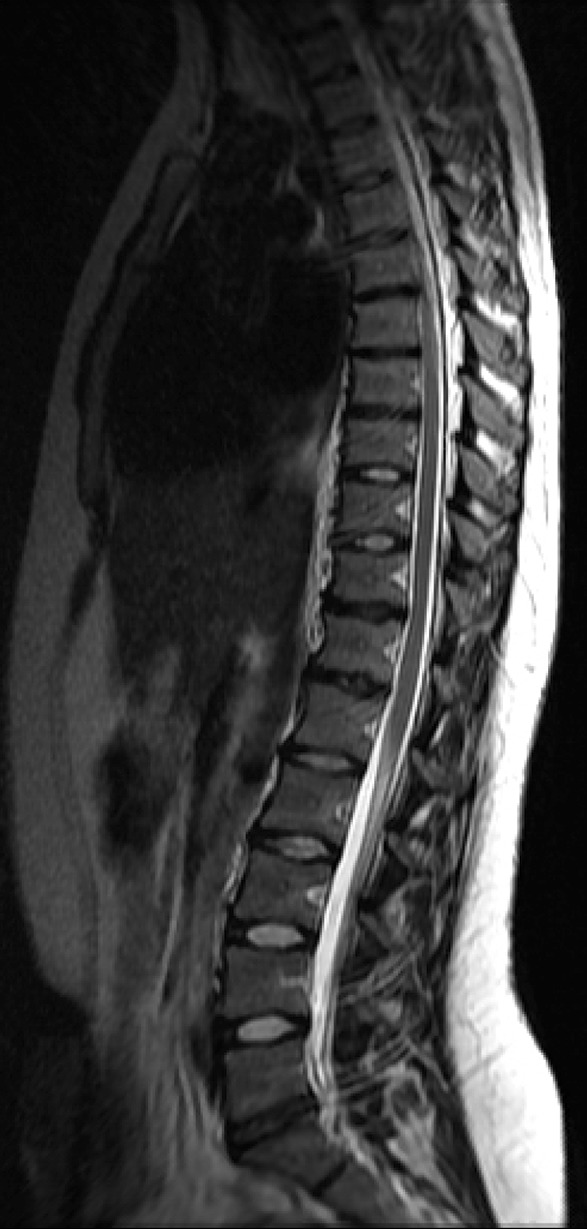
*T*_2_-weighted MRI image of the thoraco-lumbar spine of a skier showing reduction of disc signal and height at several levels, in T11–T12, T12–L1, and L5–S1

**Fig. 2:**
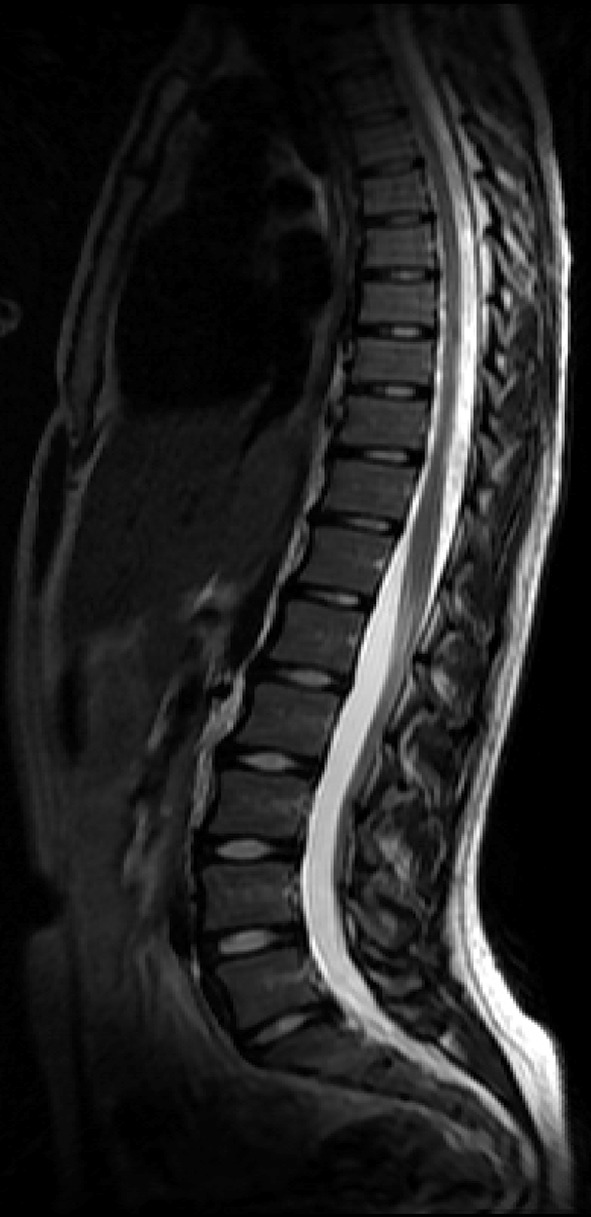
*T*_2_-weighted MRI image of the thoraco-lumbar spine of a non-athlete without any pathologies

The purpose of the present study is to identify MRI changes in the thoraco-lumbar spine and the lifetime prevalence of back pain, as well as the association between them, in young elite skiers compared to a non-athletic control group (Figs. 1, 2).

## Materials and methods

### Subjects

Seventy-five elite skiers between 16 and 20 years of age were recruited from Åre Ski Academy in Ostersund, Sweden. The skiers included both alpine (*n* = 59) and mogul skiers (*n* = 16). For comparison, a control group of non-athletic first-year high school students (*n* = 27) was recruited from Jarpen and Ostersund, Sweden.

The inclusion criteria for the skiers group were training and competing at elite level within the high school competitions. Inclusion criteria for the control group were no previous nor present participation in any organized sport activities, neither any physical activity more than 2 h per week. Participants, skiers and non-athletes, were excluded if they had had an episode of traumatic injury of the thoraco-lumbar spine or a history of previous surgery on the spine, pelvis, or hip joints. In addition, the exclusion criteria included pregnancy and any history of systemic disease including inflammatory arthritis or pelvic inflammatory disorders. The body mass index (BMI) of each participant was measured.

All participants and their parents received both written and oral information about the study.

### MRI examinations

Thoraco-lumbar spinal MRI examinations were performed at the Department of Radiology at Ostersund Hospital, Sweden with a 1.5 Tesla (GE HDXt signa echospeed) covering T5 to S3. The MRI protocol included sagittal T1 (TR560/TE < 90)- and *T*_2_ (TR4463/TE110)-weighted sequences with field of view (FOV) 48 cm and slice thickness 4 mm.

Each disc (T6–S1) was graded according to Pfirrmann classification [32]. The total number of discs graded Pfirrmann ≥3 per individual was registered, but it was enough with one disc graded Pfirrmann ≥3 to consider that individual positive for degenerative disc changes.

Each MRI was also evaluated according to a standardized protocol including disc and vertebral characteristics as previously published and summarized below [4, 5].

The evaluation of the MRI examinations was repeated after more than 1 year, blinded to the first one, to assess intra-observer reliability. To assess inter-observer agreement another radiologist evaluated the MRI examinations in 10% of subjects.

Disc signal was evaluated on *T*_2_-weighted images and graded as follows; 0 = normal, 1 = moderately reduced signal, or 2 = severely reduced signal.

Disc height reduction was defined as reduced height as compared to normal appearing discs and graded as 0 = normal, 1 = slight; reduction ≤50%, 2 = moderate; height reduction 50–90% and 3 = severe; height reduction >90%.

Disc bulging was defined as a convex extension of the disc beyond the cortex of the vertebrae and graded as 0 = normal, 1 = bulging disc.

Disc herniation was recorded only when there was a focal extrusion of the disc within the spinal canal or intervertebral foramina.

Schmorl’s nodes were defined as a clear non-marginal defect in the vertebral endplate. Smooth, shallow bulges in the endplate or minimal endplate irregularities were excluded. Schmorl’s nodes were graded as 0 = normal, 1 = slight, 2 = moderate/severe.

Abnormal configuration of the vertebral bodies was defined as anterior wedging, irregularity and flattening of the vertebral end plates, or increased antero-posterior (AP) diameter of the vertebral body as subjectively compared to the other vertebrae in the spine. It was classified as 0 = normal, 1 = wedging, 2 = flattening and 3 = increased AP diameter.

Apophyseal injuries were considered as separation of an apophyseal bony fragment from the vertebral body or deformation of the apophyseal region. These abnormalities were graded as 0 = normal, 1 = slight, 2 = moderate/severe.

Stress fractures and HIZ (high-intensity zone) were considered as high signal intensity on *T*_2_-weighted sequences in the skeleton, respectively, in the annulus fibrosus and graded as 0 = absent and 1 = present.

Spondylolisthesis was graded as 0 = absent or 1 = present. Upright whole spine plain radiographs were obtained for another study; these examinations were evaluated together with the MRI [36].

It was enough with one disc graded ≥1 according to the standardized protocol to consider that individual positive for degenerative disc changes.

Accordingly, degenerative spinal changes were graded in two different ways in which the latter include a wider spectrum of degenerative changes while the former (Pfirrmann classification) only evaluates disc signal and height.

### Back pain: questionnaires

All skiers and controls answered a questionnaire that was developed by Sward et al. [40] and Baranto et al. [5] including Visual Analogue Scale (VAS), Oswestry Disability questionnaire (ODI), and EuroQoL (EQ-5D) questionnaires [21].

Back pain was defined as existing or non-existing, present or previous pain of any kind in the thoraco-lumbar spine. The questionnaires included also subjective assessment of health status as excellent, very good, good, or poor.

Athletic and physical activity was investigated through questions about present and previous activity level. The current activity level was further categorized according to the number of training hours per week.

The study was approved by the Regional Ethical Review Board in Gothenburg at Sahlgrenska Academy, University of Gothenburg, Gothenburg, Sweden. ID: 692-13.

### Statistical analysis

The data were analysed using IBM SPSS Statistics for Windows, version 22.0. Armonk, NY: IBM Corp. The description of data was expressed in terms of mean, standard deviation (SD) and range including frequencies and percentages. For comparison of continuous variables, the Student’s independent *T* test was used. Pearson’s Chi-squared test was performed to compare the distribution of back pain between groups, and Fisher’s exact test to compare the distribution between groups when the expected cell count was less than 5. The statistical significance for all tests was set as *p* < 0.05. The Kappa statistics were used to summarize the inter- and intra-observer reliability of the ratings. The schema of Landis and Koch was used to interpret the strength of agreement [24].

## Results

MRI examinations of 65 elite skiers and 26 controls were available for the final data analysis. Ten skiers and one control were not examined due to random drop-outs and failure to attend investigations. Reasons given were difficulties with timings for MRI appointments and participants being worried about claustrophobia.

With regard to back pain questionnaires, one skier and two controls did not answer the questionnaires why 74 elite skiers and 25 controls were available for data analysis.

### Group characteristics

Demographic characteristics of the participants are summarized in Table 1. The mean age was 18.2 (SD 1.1) in skiers and 16.4 (SD 0.6) in controls. The mean body mass index (BMI) for the skiers was 22.9 (SD 2.1) and for the controls 22.7 (SD 5.3). 74% of skiers had an average 9–11 or more training hours per week compared with 78% of controls with an average 2–5 training hours per week (Table 2). 78% of skiers subjectively perceived their health as very good to excellent compared to 48% of controls (*p* = 0.026).

**Table 1:** Demographic characteristics

	Skiers (*n* = 75)	Controls (*n* = 27)
Gender, F/M %	47/53	67/33
Age, *n* (SD)	18.2 (1.13)	16.4 (0.58)
Height, cm (SD)	174 (8.20)	172 (8.57)
Weight, kg (SD)	70 (9.14)	67 (17.90)
BMI (SD)	22.9 (2.12)	22.7 (5.25)

*BMI* body mass index, *F* female, *M* male

**Table 2:** Training hours per week stratified by skiers and controls

Training hours per week	Skiers (*n* = 74)	Controls (*n* = 23)
>11 h	27 (36.5%)	0
9–11 h	28 (37.8%)	0
6–8 h	18 (24.3%)	4 (17.4%)
3–5 h	1 (1.4%)	11 (47.8%)
0–2 h	0	7 (30.4%)
0 h	0	1 (4.3%)

Chi-squared test, *p* < 0.001. Number and (%)

### Radiological findings

56% of skiers had at least one disc Pfirrmann grade ≥3 compared to 30% of controls (*p* = 0.027). Schmorl’s nodes and disc height reduction were significantly more common in skiers compared to controls, with Schmorl’s nodes seen in 46% of skiers while in 0% of controls (*p* < 0.001) and reduced disc height found in 37% of skiers compared to 0% in controls (*p* < 0.001). When all parameters in the standardized protocol were combined together, the skiers had significantly higher rate of radiological changes than controls, 82% compared to 54% (*p* = 0.007) (Table 3).

**Table 3:** MRI changes and lifetime prevalence of back pain stratified by skiers and controls

	Skiers	Controls	*p* value^a^
Pfirrmann grade ≥3	36 (56%)	8 (30%)	**0.027**
Schmorl’s nodes ≥1	30 (46%)	0 (0%)	**<0.001** ^**b**^
Disc signal reduction ≥1	45 (69%)	11 (42%)	**0.017**
Disc bulging ≥1	44 (68%)	12 (46%)	**0.036**
Disc height reduction ≥1	24 (37%)	0 (0%)	**<0.001** ^**b**^
Disc hernia ≥1	1 (2%)	0 (0%)	N/A
HIZ	5 (8%)	0 (0%)	(n.s.)
All degenerative disc changes	53 (81.5%)	14 (53.8%)	**0.007**
No degenerative disc changes	12 (18.5%)	12 (46.2%)	N/A
Lifetime prevalence of back pain	37 (50%)	11 (44%)	(n.s.)
Discs with Pfirrmann grade ≥3 per individual mean/median (range)	1.1/1 (0–6)	0.6/0 (0–3)	N/A

Bold style indicating statistical significance

*HIZ* high-intensity zone, *N/A* not analysable, violating statistical assumptions

^a^Chi-squared test

^b^Fisher’s exact test

The mean number of discs with Pfirrmann grade ≥3 was 1.1 per individual in skiers (median 1 and range 0–6) versus 0.6 in controls (median 0 and range 0–3).

Minimal or no findings were evident in both skiers and controls concerning; spondylolisthesis, HIZ, apophyseal injuries, abnormal configuration of the vertebrae, and fractures. Statistical analysis was not amenable.

There was no statistically significant difference in the rate of disc degeneration when alpine skiers (77%) were compared with mogul skiers (93%) (n.s.). The rate of Schmorl’s nodes was 42% in alpine skiers and 56% in mogul skiers without any statistically significant difference (n.s.).

### Back pain

There was no significant difference in the lifetime prevalence of back pain between skiers (50%) and controls (44%) (n.s.) nor between mogul (53%) and alpine skiers (50%). Back pain was neither correlated with age nor gender.

There was no significant difference in duration and onset of back pain between the groups (n.s.). However, a significant difference was shown for the greatest level of pain recorded during the past 6 months in the skiers (VAS 5.3, SD 3.1) compared with the controls (VAS 2.4, SD 2.0) (*p* = 0.025). Only skiers (10%) reported radiating pain to the thighs.

### Correlation between back pain and MRI findings

There was no statistical correlation between different MRI abnormalities and lifetime prevalence of back pain in skiers. Neither age nor gender correlated with the above.

### Validity

Regarding Pfirrmann classification, the study achieved an almost perfect agreement between the raters with Cohen’s kappa coefficient *κ* = 0.83. The observed agreement between the radiologists was 89% in the standardized protocol. The intra-observer reliability of the standardized protocol was substantial to almost perfect with Cohen’s kappa coefficient ranging between *κ* = 0.79 and *κ* = 1.00.

## Discussion

The most important finding of the present study was the higher rate of degenerative disc changes on MRI of the thoraco-lumbar spines of young elite skiers (56%) compared to controls (30%). Lifetime prevalence of back pain was not significantly different between the groups, 50% in skiers and 44% in controls. The radiological changes did neither correlate with back pain prevalence nor age and gender.

The reported higher rate of degenerative disc changes in the present study is in accordance with previous studies investigating spine changes in athletes practicing other sports [4, 23, 29, 33]. Kulling et al. reported in a study of professional beach volleyball players, mean age 28 years, that the prevalence of disc degeneration on MRI was 79% and spondylolysis 21% being three times higher than general population [23]. Similar results were reported in weight lifters, wrestlers, orienteers, and ice-hockey players [4]. This higher rate of degenerative changes in young elite athletes is believed to be secondary to overloading the spine with excessive lengthy training and/or probably faulty techniques [12, 22, 38]. In female gymnasts, it was found that increased intensity and length of training correlated with the rate of abnormalities seen on MRI [12]. The skiers in the present study exercise significantly more than controls as the majority (74%) exercise 9–11 h a week, while 77% of controls exercise 2–5 h a week.

The present study showed no difference in the rate of disc degenerative changes in alpine skiers compared to mogul skiers, and this possibly suggests that overloading impact on the spine may not be different between these groups.

A higher rate of spinal degenerative changes was detected in our study compared to previous studies evaluating plain radiographs [16, 29], and this is likely due to the higher sensitivity and specificity of MRI [19, 35].

The mean age difference between skiers and controls, 18.2 years (SD 1.1) and 16.4 years (SD 0.6), respectively, is something to be deliberated. Some studies have suggested a difference in the rate of radiological abnormalities and back pain prevalence during and after growth spurt, without specifying the age though [4, 37]. It was previously reported for instance that the incidence of degenerative changes tends to be higher during growth spurt [4, 5, 12, 14, 20, 34, 37, 41, 42]. However, it is uncertain whether or not this mean age difference affects the results of the present study since the fusion of vertebral growth plates has been reported to be occurring already before 17 years of age [25, 44].

The lifetime prevalence of back pain was not significantly different between the skiers and controls, which is in accordance with previous studies of alpine skiers [30, 31]. Correspondingly, a 10-year cohort study of cross-country skiers, rowers, and orienteers showed no difference of back pain prevalence compared to control subjects [11]. Meanwhile, other studies have suggested that low back pain prevalence was higher among endurance athletes with repetitive back loading [13, 29, 36, 40]. This controversy warrants further investigation, preferably with prospective studies to minimize any potential recall bias if possible. The skiers in the present study had a significantly greater level of pain recorded on VAS (VAS 5.3, SD 3.1) than controls (VAS 2.4, SD 2.0), and also only skiers (10%) reported radiating pain to the thighs. Accordingly, the skiers could have been at higher risk for nerves compression or other devastating complications. As the assessment of pain is by definition subjective, underestimating pain is possible. This is especially true considering that skiers are more familiar with pain due to trauma and injuries and seem to have higher tolerance for back pain than controls [13].

Age and gender did not appear to affect the results in the present study. Equivalent outcomes were reported when athletes were previously studied [36, 42].

There was no correlation between radiological spinal abnormalities and back pain prevalence in the present study. Several previous studies of athletes practicing other sports reached the same conclusion [4, 23, 29, 33]. One reason might be the formerly mentioned extra-spinal causes of back pain in athletes, like muscle injuries and ligaments tears [28]. Since both this study and previous studies have not evaluated both extra-spinal and spinal changes together, careful interpretation of MRI in young athletes with back pain is suggested since disc degeneration may not be the causal factors [23]. On the contrary, the association between disc degeneration and lifetime experience of low back pain was significant when baseball players and swimmers were studied [15]. Also few other studies suggested with caution, an association between disc degeneration and back pain in athletes [5, 8, 40].

78% of the skiers subjectively perceived their health as very good to excellent, while 48% of controls had similar perception. Equivalent results were reported in another study of basketball master players [27]. The latter may explain how physical activity positively correlates with the perception of health-related quality of life [3, 43].

Participation in alpine skiing at early age seems to increase the risk of developing spine abnormalities. Improved diagnostics is essential to understand the pathology and potential mechanisms of these abnormalities in order to be able to develop rehabilitation programs and prevention methods for young skiers [26].

Longitudinal studies are warranted to elucidate if the higher prevalence of degenerative disc changes in athletes will result in complications later in life.

The intention of the present study was to include age-matched groups, but some skiers had other training and competition commitments before attending the Ski Academy why the skiers were slightly older than controls. It was also difficult to find young individuals that had not taken part in any kind of training to be included within the control group.

Being an observational study, investigating all available subjects, power calculations were not tested prior to the study.

As an observational study, the presence of confounders in addition to the main exposure factor is always a possibility. We chose to include subjects from the same area in order to minimize the risk of exposure to confounders.

Another aspect is the risk for recall bias mainly concerning back pain questionnaires. These questionnaires were also patient-reported information why there might be flaws in the process. A potential limitation could possibly be a selection bias which is based on the belief that elite skiers with back pain and/or MRI abnormalities could have quit skiing due to back pain already before we started our study and therefore were not included in the study.

## Conclusion

Alpine skiers have more degenerative disc changes compared to non-athletes, but these changes do not correlate with the lifetime prevalence of back pain. The latter is not significantly different between the groups; however, skiers report more severe back pain on VAS score. Longitudinal studies to elucidate if disc degeneration in young athletes will result in complications later in life are warranted.
